# Risk screening by the emergency medical services identifies older patients at risk of emergency department readmission: a retrospective observational study

**DOI:** 10.1007/s40520-025-02942-8

**Published:** 2025-03-01

**Authors:** Eeva Saario, Marja Mäkinen, Maaret Castrén, Esa Jämsen

**Affiliations:** 1 Department of Emergency Medicine and Services, Helsinki University Hospital and University of Helsinki, Helsinki, Finland; 2 Prehospital Emergency Medical Services, Satakunta Wellbeing Services County, Pori, Finland; 3 Faculty of Medicine (Clinicum), University of Helsinki and Department of Geriatrics, Helsinki University Hospital, Helsinki, Finland

**Keywords:** Emergency medical services, Emergency departments, Risk screening, Older patients

## Abstract

**Background:**

Malnutrition, falls, and cognitive impairment are common in older patients visiting the emergency department (ED). Early recognition of these conditions could trigger interventions to improve outcomes following ED visits.

**Aim:**

To analyze whether a simple risk screening protocol in the emergency medical services (EMS) identifies older patients at risk of ED readmission.

**Methods:**

The EMS screened the falls risk, nutritional risk, and cognition of 472 patients (age ≥ 70 years) transported to the ED of a Finnish secondary care hospital between November 2018 and July 2019. Data on the risk screening, comorbidities, and ED readmissions were collected from electronic patient records. Data were analyzed using negative binomial regression, and the results are presented as incidence rate ratios (IRRs).

**Results:**

Altogether 312 patients (66%) experienced 880 ED readmissions during the 12-month follow-up. Nutritional risk was associated with an increased ED readmission rate across all time categories (< 1, 1–3, 3–6, and ≥ 6 months; IRRs 1.36–1.62, p-values < 0.05). Falls risk was associated with ED readmissions from one month after the index ED visit (IRRs 1.41–1.57, p-values < 0.05). Impaired cognition had no effect on readmissions (IRRs 1.14–1.26, p-values > 0.1).

**Conclusions:**

Patients with nutritional risk or falls risk, identified by the EMS, had a higher ED readmission rate independent of comorbidity. EMS risk screening could supplement the assessment in the ED to better identify older patients who might benefit from more detailed assessment of their health status and interventions to prevent ED readmission.

**Supplementary Information:**

The online version contains supplementary material available at 10.1007/s40520-025-02942-8.

## Introduction

The population in Finland and other western countries is aging rapidly, which is challenging health care systems [1]. The demand of emergency department (ED) services is increasing as older people visit EDs more frequently than the younger population [1–6]. Generally, in Europe, about 20–25% of patients visiting specialized medical care EDs are at least 75 years old [2, 7–9]. The demographic change also affects the prehospital emergency medical services (EMS). In Sweden, the proportion of EMS assignments involving older patients, and the overall number of EMS assignments have already been reported to be growing [10, 11] and similar developments can be expected to occur in other countries as well. Older patients have higher hospitalization rates, and they are at increased risk of adverse outcomes, such as ED readmissions and mortality [1–4, 8, 9, 12]. They also require more resources [1, 2, 6] and stay longer in EDs [4, 6], increasing pressure on already crowded EDs.

Falls, malnutrition, and impaired cognition are common, yet often unrecognized, underlying causes for older patient ED visits [3, 4, 6, 8, 12–14]. These geriatric syndromes are also associated with a higher risk of adverse outcomes, such as ED readmission [4, 15–18]. In order to reduce the burden to EDs, there is a need to find ways to decrease the number of ED visits [19]. It is widely acknowledged that those patients at high risk of adverse outcomes should be recognized, thus enabling possible interventions to prevent adverse outcomes and to decrease readmission and hospitalization rates [4, 20]. The general purpose of any screening is to identify high-risk patients who need more careful examination. In contrast, assessment is a more detailed process in which a high-risk patient is diagnosed with a certain problem, and suitable tailored interventions to enhance the situation are recommended [21–23]. Since June 2018, screening older patients for falls risk, nutritional risk, and impaired cognition has been determined as a routine procedure in the EMS in Espoo region, Finland. The EMS aims to identify patients at risk and pass the information to the EDs for more comprehensive assessment. Most screening tools targeted for ED use are quite general and aimed for identifying patient in need for more comprehensive assessment, whereas here the aim was to identify risks that the ED and hospital staff could directly act on. The EMS had not performed such risk screening before. Our prior study showed that the prevalence of high-risk patients in the EMS risk screening corresponded to risk screening results in ED settings [24]. 

The aim of this study was to observe whether screening of falls risk, nutritional risk, and impaired cognition by the EMS could help to identify older patients at high risk of ED readmission.

## Methods

### Setting and participants

The EMS performed a risk screening of community-dwelling patients who were at least 70 years old and non-urgently transported with an ambulance to the ED of a large secondary care hospital in Espoo (population ca. 300,000), Finland, between 10 November 2018 and 30 July 2019. Patients living in nursing homes, patients with an acute health condition requiring treatment by the EMS, and patients whose general condition hindered them from answering the risk screening questions were not screened. All other patients meeting the criteria were eligible for screening, regardless of their illnesses or comorbidities. The detailed data collection has been reported previously [24].

### Risk screening in the EMS

**Fig. 1:**
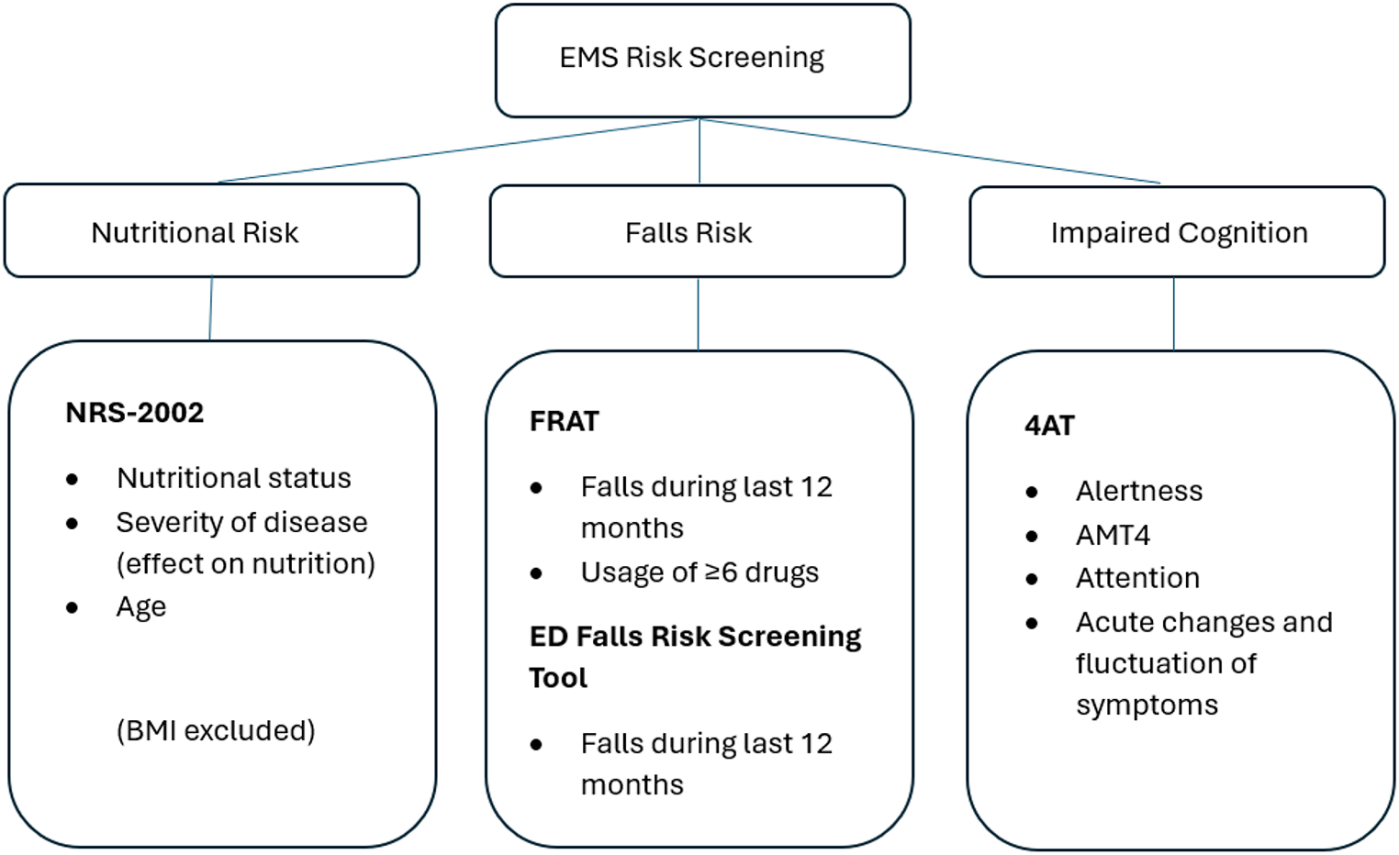
Risk screening tool in the EMS. EMS = Emergency medical services, NRS-2002 = Nutritional Risk Screening 2002, BMI = Body mass index, FRAT = Falls Risk Assessment Tool, ED = Emergency department, 4AT = the 4 ‘A’s Test, AMT4 = Abbreviated Mental Test

In Finland, the role of the EMS is to assess the need for treatment, recognize patients with acute illnesses, provide urgent treatment, and to transport patients to EDs [25, 26]. At the time of the study, the EMS in the Espoo area also performed risk screening on older patients [24]. The risk screening of older patients in the EMS setting consisted of three parts: screening of nutritional risk, falls risk, and impaired cognition (Fig. 1). The EMS used selected pre-existing screening tools that had been pre-validated to in-hospital use but not for EMS setting. The ED where the EMS transported the patients to, did not routinely use any risk screening protocol or instruments. The Nutritional Risk Screening 2002 (NRS-2002) tool is a widely used, rapidly administrated tool for screening the risk of malnutrition [27, 28]. Falls risk was screened by the ED falls risk screening tool [29] that was supplemented by selected parts of the Falls Risk Assessment Tool (FRAT) [30]. The 4 ‘A’s test (4AT) was used in screening of impaired cognition [31]. Scoring ≥ 3 points in any of the risk screenings (nutritional risk, falls risk, or impaired cognition) was considered a positive screening result, i.e., the patient was categorized as a risk patient of that specific matter. The risk screening tools were accessible to the EMS in the real-time electrical reporting and management program (Merlot-Medi^®^, CGI Suomi Oy, Helsinki, Finland) routinely used during all EMS calls.

### ED readmissions

Data on ED readmissions to any of the eight adult ED units of Helsinki University Hospital within 12 months of the initial ED admission were manually obtained from the electronic patient reports. The data included secondary- and tertiary-care-level ED readmissions. Contacts to primary care were not included. ED readmissions during the 12-month period were recorded in four categories based on readmission time: less than one month, one month up to under three months, three months up to under six months, and six to twelve months from the initial ED visit. The ED readmission reasons were divided into four categories: readmissions due to falls, decreased general condition, confusion, or other reasons.

EMS risk screening data were supplemented by diverse information collected from patient records. Reasons for ED admissions were listed based on the first reason reported as the patient entered the ED. Patients’ comorbidities were recorded following the list of comorbidities included in the Charlson Comorbidity Index (CCI) [32]. Any mentions of home care services, need for assistance in daily activities, and need for mobility aid were observed but because of high proportion of missing or inaccurate data this information could not be used in the analyses. Furthermore, EMS re-encounters with the same patients and mortality during the 12-month period were recorded. All data on initial ED admissions, readmissions, mortality, and EMS re-encounters were collected manually from the electronic patient reports (Uranus^®^, CGI Suomi Oy, Helsinki, Finland, and Apotti^®^, Oy Apotti Ab, Helsinki, Finland).

### Statistics

All categorical data are represented as counts with percentages in parentheses (%) and all continuous variables with medians and interquartile ranges (IQRs). Negative binomial regression was used to model variables associated with ED readmissions, and to take into account accumulation of ED visits during the follow-up. The models were also adjusted for age, sex, comorbidities, and the risks (falls risk, nutritional risk, impaired cognition). The results are reported as incidence rate ratios (IRR) with 95% confidence intervals. The Mann-Whitney U test was used for pairwise comparison of variables. All p-values below 0.05 were considered significant. Analyses were done with SPSS version 28 and R software version 4.3.2 using package MASS for fitting negative binomial regression models.

## Results

### Description of the study sample

During the data collection, 11/2018-07/2019, the EMS transported 5792 patients aged ≥ 70 years non-urgently to the ED. The EMS performed the risk screening on 472 patients (8%). The mean age of the patients was 82.8 years (IQR 77.5–87.9, range 70.2–103.7). Most of them were female (*n* = 293, 62%) and were receiving home care services (*n* = 336, 71%). Out of the patients, 154 (33%) had one, 159 (34%) had two, 54 (11%) had three, 29 (6%) had four, and 7 patients (1%) had five comorbidities. Sixty-nine patients (15%) did not have any recorded comorbidity. The most common causes for initial ED visits were falls (*n* = 139, 29%), decreased general condition (*n* = 103, 22%), and chest pain (*n* = 41, 9%). One third of patients (*n* = 155, 33%) were discharged home from the ED, and 311 patients (66%) were transferred to hospital wards for further treatment. Seven patients (1%) were transferred to hospices for palliative care. None of the patients died during the initial visit to the ED, but the mortality during the 12-month follow-up was 21% (*n* = 97). The EMS identified falls risk in 209 (43%), nutritional risk in 81 (17%), and impaired cognition in 134 (28%) patients. Of all patients screened, 187 (40%) had no risks at all, 173 (37%) had one identified risk, 92 (20%) had two risks, and 20 patients (4%) had all three risks simultaneously.

### Readmissions

**Fig. 2:**
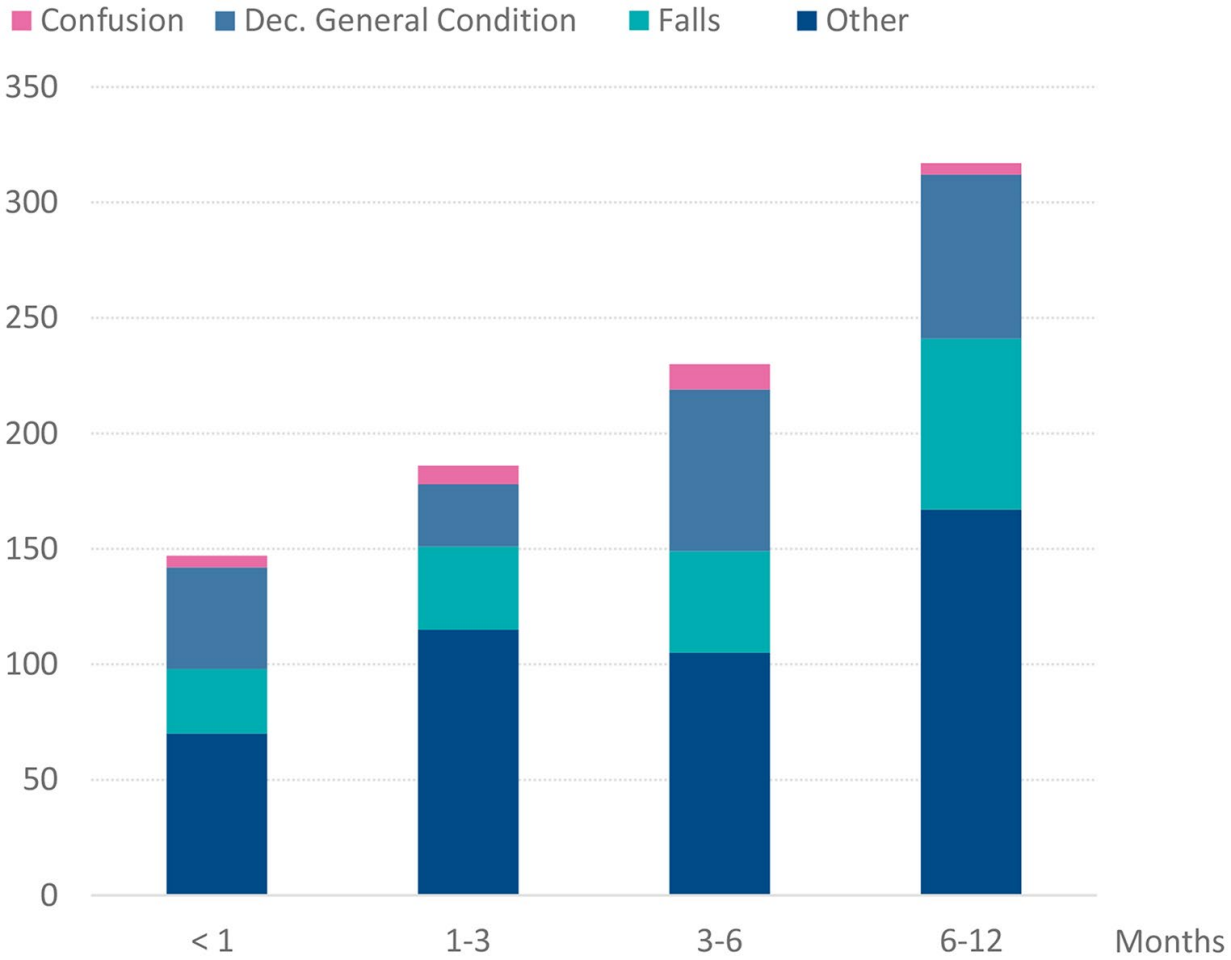
The numbers and causes of ED readmissions (*n* = 880) during different phases of the 12-month observation period

Within 12 months of the initial ED visit, 312 patients (66%) had a combined total of 880 ED readmissions (Fig. 2). There were 81 patients (17%) with four or more ED readmissions. The highest number of ED readmissions, seen in two patients, was 12. Readmission rates were 23% at one month, 42% at three months, 54% at six months, and 66% at 12 months. Falls and decreased general condition accounted for 21% (*n* = 182) and 24% (*n* = 272) of ED readmissions, respectively (Fig. 2). During the 12-month follow-up period, the EMS encountered the same patients 1043 times. In 761 of these events (73%), the patient was readmitted to the ED.

### Association of identified risks with readmissions

Based on the negative binomial regression (Table 1), nutritional risk was associated with an increased rate of ED readmission in all time categories (IRRs 1.36–1.62, p-values < 0.05) and falls risk after one month (IRRs 1.41–1.57, p-values < 0.02). Furthermore, comorbidities increased ED readmissions after one month (IRRs 1.17–1.22, p-values < 0.001–0.0096). Neither impaired cognition, age, nor sex were related to ED readmissions. In multivariable analysis adjusted for age, sex, number of comorbidities, and identified risks, nutritional risk and falls risk remained statistically significant throughout almost the entire follow-up period. Patients with comorbidities also more likely had an ED readmission after one month (IRRs 1.18–1.24, p-values < 0.01).

**Table 1:** Association of identified risks with all ED readmissions during different phases of the 12-month follow-up period

	< 1 month		1–3 months		3–6 months		6–12 months	
Univariate analysis	IRR (95% CI)	*p*-value	IRR (95% CI)	*p*-value	IRR (95% CI)	*p*-value	IRR (95% CI)	*p*-value
Falls risk	1.32 (0.90 − 1.93)	0.15	1.41 (1.07–1.88)	0.016	1.48 (1.16–1.89)	0.0016	1.57 (1.26–1.95)	< 0.001
Nutritional risk	1.62 (1.03–2.54)	0.035	1.56 (1.10–2.22)	0.012	1.38 (1.01–1.89)	0.044	1.36 (1.03–1.80)	0.033
Impaired cognition	1.19 (0.78–1.78)	0.42	1.26 (0.93–1.71)	0.14	1.17 (0.89–1.53)	0.26	1.14 (0.90–1.45)	0.29
Age	1.00 (0.97–1.03)	0.98	1.02 (1.00–1.04)	0.12	1.01 (0.99–1.03)	0.31	1.01 (0.99–1.02)	0.41
Sex	1.23 (0.83–1.80)	0.30	1.16 (0.87–1.54)	0.33	1.23 (0.96–1.59)	0.10	1.14 (0.91–1.43)	0.26
Number of comorbidities	1.16 (0.99–1.37)	0.064	1.17 (1.04–1.33)	0.0096	1.19 (1.07–1.33)	0.0012	1.22 (1.12–1.34)	< 0.001
**Multivariable ** analysis*								
Falls risk	1.26 (0.85–1.85)	0.24	1.31 (0.99–1.75)	0.060	1.44 (1.13–1.85)	0.0035	1.58 (1.27–1.96)	< 0.001
Nutritional risk	1.55 (0.97–2.44)	0.061	1.50 (1.06–2.13)	0.023	1.31 (0.96–1.80)	0.085	1.29 (0.98–1.70)	0.071
Impaired cognition	1.07 (0.70–1.62)	0.75	1.15 (0.84–1.57)	0.38	1.04 (0.79–1.36)	0.79	0.99 (0.78–1.25)	0.90
Age	1.00 (0.97–1.03)	0.87	1.02 (1.00–1.04)	0.092	1.01 (0.99–1.03)	0.26	1.01 (0.99–1.02)	0.43
Sex	1.14 (0.77–1.68)	0.52	1.10 (0.83–1.47)	0.52	1.17 (0.91–1.50)	0.23	1.07 (0.85–1.33)	0.57
Number of comorbidities	1.16 (0.99–1.38)	0.065	1.18 (1.05–1.34)	0.0062	1.20 (1.08–1.34)	< 0.001	1.24 (1.13–1.36)	< 0.001

*Variables adjusted by age, sex, comorbidities, and identified risks. IRR = incident risk ratio, CI = confidence interval

Supplementary figures describe the associations between age, sex, number of comorbidities, and identified risks with ED readmissions due to different reasons. After one month, falls risk was associated with ED readmission due to falls (IRRs 2.74–3.82, p-values < 0.001) and decreased general condition (IRRs 1.75–2.00, p-values < 0.001–0.015). Falls risk was associated with confusion-related ED readmissions after six months (IRR 2.19, 95% CI 1.02–4.88, p-value < 0.05). Nutritional risk increased fall-related ED readmission rates in all time categories (IRRs 1.83–6.44, p-values < 0.001–0.013), particularly during the first month (IRR 6.44, 95% CI 2.55–16.92, p-value < 0.001). It was also associated with ED readmissions due to decreased general condition after six months (IRR 1.65, 95% CI 1.08–2.52, p-value 0.021). Identified risks were not associated with ED readmissions where the reason was categorized as “other”, but number of comorbidities was after one month (IRRs 1.24–1.30, p-values < 0.001–0.0087). Comorbidities were also associated with ED readmissions due to decreased general condition (IRRs 1.28–1.56, p-values < 0.05) throughout the follow-up period. Finally, male sex was associated with a higher risk of fall-related ED readmission (IRRs 1.57–2.95 p-values < 0.05). Most of these differences remained statistically significant after adjustments but IRRs could not be calculated for all time periods due to insufficient number of cases. Age had no effect in any of the adjusted analyses.

Altogether, 81 of the patients experienced ≥ 4 ED readmissions during the follow-up. Compared to patients with < 4 readmissions, they had more chronic diseases and they were more often at falls risk (57% vs. 40%, *p* = 0.01) but there were no differences in age, sex or other risks.

## Discussion

This study investigated whether a simple risk screening protocol in the EMS can identify older patients at high risk of ED readmission over a 12-month follow-up period. Indeed, patients with identified nutritional risk had an increased rate of ED readmission for all recorded reasons throughout the 12-month follow-up period. Furthermore, falls risk was associated with ED readmission after one month. Both falls risk and nutritional risk also increased fall-related ED readmission and falls risk also increased readmission related to decreased general condition. On the other hand, ED readmissions occurring due to decreased general condition or other reasons than falls were more evidently associated with comorbidities. Therefore, the results show that the EMS risk screening tool is able to not only identify patients with ED readmission risk, but also distinguish patient groups based on predictable reasons for ED readmission. Overall, the risks were common in screened patients as more than half of the patients (60%) had at least one identified risk, and almost one fourth of the patients (24%) had several risks simultaneously.

During the 12-month follow-up, which was further divided to four time categories based on prior studies [2, 5–7, 9, 12, 15, 18, 33–39], most of the screened patients (66%) experienced at least one ED readmission, and many experienced several ED visits, emphasizing the importance of considering the accumulation of ED visits instead of just focusing on first readmissions. The observed ED readmission rates after one month (23%) and three months (42%) are within the range reported in a prior systematic review (10.3–37.6% and 16–58%, respectively) [33]. A 12-month readmission rate is not often reported, but in a single earlier study [7] it was lower (42%) than in our study (66%), probably because our data covered readmissions to other units in the area too and included also persons discharged home from the initial ED visit. Therefore, the data included readmissions to EDs with certain specialties, such as trauma care. During the follow-up period, the EMS re-encountered the patients for over 1000 times. Although the patient was usually also readmitted to an ED, in about a quarter of cases, the patients stayed on site after the EMS had assessed the need for ED care. For those patients, the EMS remained the only acute health care contact at that time. Altogether, the present results demonstrate the burden of accumulation of ED visits and an even greater burden to the EMS.

The univariate analysis showed that the ED readmission rate was higher among those patients the EMS had recognized as being at nutritional or falls risk during screening. In adjusted multivariable analysis, some of the differences between variables were marginally non-significant. The IRRs, however, remained constant, suggesting that the non-significant results may be due to low statistical power rather than true non-significance. The results are in line with prior studies stating that falls [34] and malnutrition [12] are associated with poor outcomes, such as readmission, after an ED visit.

It is acknowledged that cognitive impairment predisposes a patient to ED readmission [7, 15, 16, 35]. However, in our study, impaired cognition did not show an association with ED readmission. Reasons for this can only be speculated. Firstly, in our study, scoring at least three points on the 4AT was considered impaired cognition. Generally, scoring three points would be considered potential cognitive impairment (without delirium) with a more detailed assessment suggested [36]. As no further assessment was performed to the study population, the prevalence of actual cognitive impairment among patients remained unclear. Moreover, a fair proportion of delirium cases develop later during the course of the acute disease and could not therefore have been identified by the EMS. Secondly, it could be that recognizing mildly to moderately impaired cognition was difficult for the EMS.

Falls and decreased general condition are known to be common health problems in older ED patients [17, 37]. Altogether, falls and decreased general condition were major causes of ED admission and readmission. It is known that malnutrition exposes patients to falls [4], and that a history of falls predicts future falls [37, 38]. In this study, nutritional risk also predisposed patients to fall-related ED readmissions, especially early readmissions. Malnutrition may lead to sarcopenia, which again is associated with falls [39, 40]. Possible sarcopenia was not assessed, but it might partly explain at least some of the falls and fall-related ED readmissions. In order to prevent adverse effects of malnutrition and falls, it is important to identify patients at risk of such problems and to initiate suitable interventions (e.g., appointment with geriatrician, physical therapist or dietician; engaging walking aids) [37, 38, 41].

In this study, comorbidities were observed based on the list of illnesses included in the CCI [32]. Underlying comorbidities are important factors both causing and predicting ED readmissions. For example, organ failure and cancer increase the need for ED care [42]. Many chronic conditions are also risk factors for malnutrition [14]. Furthermore, polypharmacy [43], functional impairment, and increased morbidity, for example, are known to predispose patients to falls [44]. In this study, comorbidities were associated with higher ED readmission rates. Most clearly, they were associated with ED readmissions due to decreased general condition. This finding was not surprising, as it can be presumed that negative changes in comorbidities decrease overall functional ability, forcing patients to go to EDs with non-specific complaints, the admission reason thus being reported as decreased general condition. The number of comorbidities was also associated with ED readmissions due to “other reasons”, which is most likely also connected with negative changes in comorbidities. Remarkably, the number of comorbidities and fall-related ED readmissions were not associated. This finding suggests that the EMS risk screening was able to identify patients at higher risk of fall-related ED readmission, independent of comorbidities.

Almost one fifth of patients had four or more ED readmissions within the one-year period, thus being generally considered as frequent users. Frequent users have been addressed [45] as one factor leading to a common phenomenon, ED crowding. Repeat readmissions could possibly be reduced if patients at increased readmission risk were identified [45]. In our study, more than half of the frequent users were at falls risk. On the other hand, chronic diseases were more common in frequent users, suggesting that repeat use may also be related to chronic diseases.

Geriatric syndromes, such as malnutrition, falls, and cognitive impairment, are common in older patients. Yet, they are often underlying problems, while patients attend EDs for various reasons. Unfortunately, the geriatric syndromes often remain unrecognized, and patients are left without suitable interventions [1, 17, 46]. In this study, 43% of patients were at risk of fall, 17% at nutritional risk, and 28% had impaired cognition. Depression, sarcopenia, or frailty, often also present among older ED patients [8, 44, 47], were not screened. Failure to recognize geriatric syndromes potentially leads to high ED readmission risk. However, practice has shown that due to lack of time and staff resources, patients are often discharged from the ED without any risk assessment. On the other hand, comprehensive health assessment of an older patient can be rather complex, and few EDs are staffed with persons with geriatric expertise. Hence, EDs might not even be the best places to routinely perform detailed assessment [4]. EMS risk screening appears to be a feasible way to recognize risk patients [24] and estimate the risk of possible negative outcomes.

The EMS screened older patients for nutritional risk, falls risk, and impaired cognition by using selected screening tools that had been pre-validated in ED setting. The EMS risk screening protocol could be compared also to other rapidly administered screening tools commonly used in EDs, such as Identification of Seniors At Risk (ISAR) [48] or the Triage Risk Screening Tool (TRST) [49]. ISAR and TRST were created to early detect patients, who could potentially benefit from more comprehensive assessment of health problems to prevent adverse outcomes. In contrast, the screening protocol in our study consists of specific tests, which identify the patients at a certain risk, enabling immediate treatment plans and interventions to those health problems. Moreover, the EMS risk screening provides valuable information that can potentially impact patients’ treatment plans without any need for further assessments. This is especially valuable in EDs with no geriatric services and when there is a lack of time and resources to perform detailed assessments beyond management the acute condition.

The results presented here suggest that establishing a care pathway for older patients, in which EMS risk screening could be one link to intervene and improve the overall health of older patients, might be worth considering. The EMS could provide crucial information to other professionals, and the patients could be referred to suitable appointments, for example in primary care. Prior studies have stated that patients often consent to being contacted at home to discuss their health-related service needs [14] or prefer a primary care appointment over an ED admission [50]. With a proper care pathway, EMS risk screening could potentially be used to link patients to suitable authorities.

To our knowledge, this was the first time the EMS has performed such risk screening to older patients. In our prior study [24] we have reported that the EMS risk screening is feasible. Now, in this study, our findings confirm that risk screening can identify patients at risk of ED readmissions. Further research is needed on the validity of the EMS risk screening. The EMS risk screening results should be compared to results of similar screening performed in the ED by a geriatric team. Furthermore, research is needed to detect the most efficient interventions for identified risk patients.

### Strengths and limitations

This study has some particular strengths and limitations. The study was conducted in one region only, possibly limiting the generalizability of results. The screening tools are designed for in-hospital use, and they were not pre-validated for EMS use. The patient population appeared to be heterogeneous e.g., in terms of age and reasons for ED visits. The data covered all readmissions to any secondary and tertiary care EDs in the area. Therefore, this study was able to detect all possible ED readmissions, independent of readmission reasons. The risk screening was a new task for the EMS, so it is possible that the EMS personnel were not fully accustomed to performing the risk screening, which might have led to underdetection of underlying geriatric syndromes. However, the proportions of patients with identified risks corresponded to prior studies [24]. Therefore, the data were likely a quite reliable representation of the population. Unfortunately, the number of patients who met the different exclusion criteria from the total number of the patients transported by the EMS during the observation period could not be explicitly detected. This is because of limitations of the EMS task records and dispatch codes. The codes are the same for community-dwelling patients and patients living in nursing homes. Furthermore, the coding does reveal neither patients’ general condition nor possible actions of treatment during the transportation. Due to the rather low number of participants, there is reason to think that some of the non-significant results presented could be explained by lower statistical power rather than true non-significance.

The retrospective nature of the study set some limitations to data collection. Unfortunately, identifying frailty among participants was not possible, as the EMS risk screening tool did not include any frailty screening tool. Yet, frailty is widely present in older ED patients [8, 46] and is also associated with adverse outcomes after ED admission [12]. Furthermore, patients’ functional ability could not be determined. The original plan was to report the usage of, for example, walking aids and need for assistance in ADL or IADL functions. However, it became evident that those factors were poorly reported in patient records, making the potential results highly unreliable. Therefore, we had to exclude functional ability from the final data.

## Conclusions

Patients at risk of fall or nutritional risk identified by the EMS had higher rates of ED readmission compared to patients without such risks. The risk screening – together with data about comorbidities – helped to stratify the patients according to the reasons for future ED admissions. The EMS risk-screening results and other valuable information provided by the EMS could be used to supplement assessment in the ED to identify those older patients who might benefit from a more detailed assessment of their health status, referrals to specialists or other interventions, to enhance their health and coping at home, or prevent adverse outcomes.

## Electronic supplementary material

Below is the link to the electronic supplementary material.

Supplementary Material 1

## Data Availability

No datasets were generated or analysed during the current study.

## References

[1] Ukkonen M, Jämsen E, Zeitlin R, Pauniaho SL (2019) Emergency department visits in older patients: a population-based survey. BMC Emerg Med 19:20 30813898 10.1186/s12873-019-0236-3 PMC6391758

[2] Ginsburg AD, Oliveira J, e Silva L, Mullan A, Mhayamaguru KM, Bower S, Jeffery MM et al (2021) Should age be incorporated into the adult triage algorithm in the emergency department? Am J Emerg Med 46:508–514 33191046 10.1016/j.ajem.2020.10.075

[3] Deschodt M, Devriendt E, Sabbe M, Knockaert D, Deboutte P, Boonen S et al (2015) Characteristics of older adults admitted to the emergency department (ED) and their risk factors for ED readmission based on comprehensive geriatric assessment: a prospective cohort study. BMC Geriatr 15:54 25928799 10.1186/s12877-015-0055-7 PMC4417280

[4] Huang HH, Chia-Yu Chang J, Tseng CC, Yang YJ, Fan JS, Chen YC et al (2021) Comprehensive geriatric assessment in the emergency department for the prediction of readmission among older patients: a 3-month follow-up study. Arch Gerontol Geriatr 92:104255 32966944 10.1016/j.archger.2020.104255

[5] Carpenter CR, Shelton E, Fowler S, Suffoletto B, Platts-Mills TF, Rothman RE et al (2015) Risk factors and screening instruments to predict adverse outcomes for undifferentiated older emergency department patients: a systematic review and meta-analysis. Acad Emerg Med 22(1):1–21 25565487 10.1111/acem.12569

[6] Di Prazza A, Canino B, Barbagallo M, Veronese N (2023) The importance of prognosis in geriatric patients attending the emergency department: a comparison between two common short geriatric assessment tools. Aging Clin Exp Res 35(12):3041–3046 37932645 10.1007/s40520-023-02603-8 PMC10721668

[7] Launay CP, de Decker L, Kabeshova A, Annweiler C, Beauchet O (2016) Risk of unplanned emergency department readmission after an acute-care hospital discharge. J Nutr Health Aging 20(2):210–21726812519 10.1007/s12603-015-0624-7

[8] McCabe JJ, Kennelly SP (2015) Acute care of older patients in the emergency department: strategies to improve patient outcomes. Open Access Emerg Med 7:45–54 27147890 10.2147/OAEM.S69974 PMC4806806

[9] Heeren P, Devriendt E, Fieuws S, Wellens NIH, Deschodt M, Flamaing J et al (2019) Unplanned readmission prevention by a geriatric emergency network for transitional care (URGENT): a prospective before-after study. BMC Geriatr.;19(1) 10.1186/s12877-019-1233-9 PMC6686568 31390994

[10] Svensson A, Bremer A, Rantala A, Andersson H, Scott I, Facpara D (2022) Ambulance clinicians’ attitudes to older patients’ self-determination when the patient has impaired decision-making ability: a Delphi study. Int J Older People Nurs.;17 10.1111/opn.12423 34510764

[11] Rantala A, Sterner A, Frank C, Heinrich E, Holmberg B (2023) Older patients’ perceptions of the Swedish ambulance service: a qualitative exploratory study. Australas Emerg Care 26(3):249–253 36764911 10.1016/j.auec.2023.01.005

[12] Costa AP, Hirdes JP, Heckman GA, Dey AB, Jonsson PV, Lakhan P et al (2014) Geriatric syndromes predict postdischarge outcomes among older emergency department patients: findings from the interRAI multinational emergency department study. Acad Emerg Med 21(4):422–433 24730405 10.1111/acem.12353

[13] Gray LC, Peel NM, Costa AP, Burkett E, Dey AB, Jonsson PV et al (2013) Profiles of older patients in the emergency department: findings from the interrai multinational emergency department study. Ann Emerg Med 62(5):467–474 23809229 10.1016/j.annemergmed.2013.05.008

[14] Aylward AF, Engelberg Anderson J, Morris A, Bush M, Schmitthenner B, Shams R, Bin et al (2021) Using malnutrition and food insecurity screening to identify broader health-related social needs amongst older adults receiving emergency department care in the Southeastern United States: a cross-sectional study. Health Soc Care Community 29(6):e420–e430 33825280 10.1111/hsc.13367 PMC10231411

[15] Shah MN, Jacobsohn GC, Jones CMC, Green RK, Caprio TV, Cochran AL et al (2022) Care transitions intervention reduces ED revisits in cognitively impaired patients. Alzheimer’s Dement 8(1):e12261 10.1002/trc2.12261 PMC8919246 35310533

[16] Seidenfeld J, Dalton A, Vashi AA (2023) Emergency department utilization and presenting chief complaints by veterans living with dementia. Acad Emerg Med 30(4):331–339 36757144 10.1111/acem.14686 PMC10192204

[17] Tanderup A, Lassen AT, Rosholm JU, Ryg J (2018) Disability and morbidity among older patients in the emergency department: a Danish population-based cohort study. BMJ Open.;8(12) 10.1136/bmjopen-2018-023803 PMC6303572 30552269

[18] Griffin A, O’neill A, O’connor M, Ryan D, Tierney A, Galvin R (2020) The prevalence of malnutrition and impact on patient outcomes among older adults presenting at an Irish emergency department: a secondary analysis of the OPTI-MEND trial. BMC Geriatr 20:455 33160319 10.1186/s12877-020-01852-w PMC7648316

[19] Lobachova L, Brown DFM, Sinclair J, Chang Y, Thielker KZ, Nagurney JT (2014) Patient and provider perceptions of why patients seek care in emergency departments. J Emerg Med 46(1):104–112 24063881 10.1016/j.jemermed.2013.04.063

[20] Hastings SN, Horney C, Landerman LR, Sanders LL, Hocker MB, Schmader KE (2010) Exploring patterns of health service use in older emergency department patients. Acad Emerg Med 17(10):1086–1092 21040110 10.1111/j.1553-2712.2010.00870.x

[21] Reber E, Gomes F, Vasiloglou MF, Schuetz P, Stanga Z (2019) Nutritional Risk Screening and Assessment. J Clin Med 8(7):1065 31330781 10.3390/jcm8071065 PMC6679209

[22] Field LB, Hand RK (2015) Differentiating Malnutrition Screening and Assessment: a Nutrition Care process perspective. J Acad Nutr Diet 115(5):824–828 25582410 10.1016/j.jand.2014.11.010

[23] Norman KJ, Hirdes JP (2020) Evaluation of the predictive accuracy of the interRAI Falls Clinical Assessment Protocol, Scott Fall Risk Screen, and a supplementary Falls Risk Assessment Tool used in residential long-term care: a retrospective cohort study. Can J Aging 39(4):521–532 32172692 10.1017/S0714980820000021

[24] Saario EL, Mäkinen MT, Jämsen ERK, Nikander P, Castrén MK (2021) Screening of community-dwelling older patients by the emergency medical services: an observational retrospective registry study. Int Emerg Nurs 59:101078 34571450 10.1016/j.ienj.2021.101078

[25] Health Care Act 1326/2010 Finland: Finlex. Available from: https://finlex.fi/fi/laki/ajantasa/2010/20101326#L4P39

[26] Heinonen K, Puolakka T, Salmi H, Boyd J, Laiho M, Porthan K et al (2022) Ambulance crew-initiated non-conveyance in the Helsinki EMS system-A retrospective cohort study. Acta Anaesthesiol Scand 66:625–633 35170028 10.1111/aas.14049 PMC9544076

[27] Rabito EI, Marcadenti A, Da Silva Fink J, Figueira L, Silva FM, Nutritional Risk S (2002) Short Nutritional Assessment Questionnaire, Malnutrition Screening Tool, and Malnutrition Universal Screening Tool Are Good Predictors of Nutrition Risk in an Emergency Service. Nutr Clin Pract. 2017;32(4):526–32 10.1177/0884533617692527 28199797

[28] Kondrup J, Ramussen HH, Hamberg O, Stanga Z, Camilo M, Richardson R et al (2003) Nutritional risk screening (NRS 2002): a new method based on an analysis of controlled clinical trials. Clin Nutr 22(3):321–336 12765673 10.1016/s0261-5614(02)00214-5

[29] Tiedemann A, Sherrington C, Orr T, Hallen J, Lewis D, Kelly A et al (2013) Identifying older people at high risk of future falls: Development and validation of a screening tool for use in emergency departments. Emerg Med J 30(11):918–922 23139096 10.1136/emermed-2012-201783

[30] Stapleton C, Hough P, Oldmeadow L, Bull K, Hill K, Greenwood K (2009) Four-item fall risk screening tool for subacute and residential aged care: the first step in fall prevention. Australas J Ageing 28(3):139–143 19845654 10.1111/j.1741-6612.2009.00375.x

[31] O’Sullivan D, Brady N, Manning E, O’Shea E, O’Grady S, O’Regan N et al (2018) Validation of the 6-Item cognitive impairment test and the 4AT test for combined delirium and dementia screening in older Emergency Department attendees. Age Ageing 47(1):61–68 28985260 10.1093/ageing/afx149 PMC5860384

[32] Charlson ME, Pompei P, Ales KL, MacKenzie CR (1987) A new method of classifying prognostic comorbidity in longitudinal studies: development and validation. J Chronic Dis 40(5):373–383 3558716 10.1016/0021-9681(87)90171-8

[33] Cilla F, Sabione I, D’Amelio P (2023) Risk factors for early hospital readmission in geriatric patients: a systematic review. Int J Environ Res Public Health 20:1674 36767038 10.3390/ijerph20031674 PMC9914102

[34] Hoffman GJ, Liu H, Alexander NB, Tinetti M, Braun TM, Min LC (2019) Posthospital Fall Injuries and 30-Day readmissions in adults 65 years and older. JAMA Netw Open 2(5):e194276 31125100 10.1001/jamanetworkopen.2019.4276 PMC6632136

[35] Gettel CJ, Falvey JR, Gifford AM, Hoang LB, Christensen LA, Hwang U et al (2022) Emergency Department Care Transitions for patients with cognitive impairment: a scoping review. JAMDA 23:1313e1–1313e13 10.1016/j.jamda.2022.01.076 PMC9378565 35247358

[36] Bellelli G, Morandi A, Davis DHJ, Mazzola P, Turco R, Gentile S et al (2014) Validation of the 4AT, a new instrument for rapid delirium screening: a study in 234 hospitalised older people. Age Ageing 43:496–502 24590568 10.1093/ageing/afu021 PMC4066613

[37] Davenport K, Alazemi M, Sri-On J, Liu S (2020) Missed opportunities to diagnose and intervene in modifiable risk factors for older Emergency Department patients presenting after a fall. Ann Emerg Med 76(6):730–738 33010956 10.1016/j.annemergmed.2020.06.020

[38] Soukola SK, Jämsen ERK, Pauniaho S-LK, Ukkonen MT (1999) A population-based study of 2347 fall-related injuries among older people in a Finnish emergency department. Eur Geriatr Med 11:315–320 10.1007/s41999-020-00288-0 32297195

[39] Schneider DA, Trence DL (2019) Possible role of Nutrition in Prevention of Sarcopenia and Falls. Endocr Pract 25(11):1184–1190 31412231 10.4158/EP-2019-0144

[40] Landi F, Liperoti R, Russo A, Giovannini S, Tosato M, Capoluongo E et al (2012) Sarcopenia as a risk factor for falls in elderly individuals: results from the ilSIRENTE study. Clin Nutr 31(5):652–658 22414775 10.1016/j.clnu.2012.02.007

[41] Mikolaizak AS, Simpson PM, Tiedemann A, Lord SR, Caplan GA, Bendall JC et al (2013) Intervention to prevent further falls in older people who call an ambulance as a result of a fall: a protocol for the iPREFER randomised controlled trial. BMC Health Serv Res 13(1):1–8 24070456 10.1186/1472-6963-13-360 PMC3849451

[42] Wang CL, Ding ST, Hsieh MJ, Shu CC, Hsu NC, Lin YF et al (2016) Factors associated with emergency department visit within 30 days after discharge. BMC Health Serv Res 16:190 27225191 10.1186/s12913-016-1439-x PMC4879744

[43] Ozkok S, Aydin CO, Sacar DE, Catikkas NM, Erdogan T, Kilic C et al (2022) Associations between polypharmacy and physical performance measures in older adults. Arch Gerontol Geriatr 98:104553 34653922 10.1016/j.archger.2021.104553

[44] Yeung SS, Reijnierse EM, Pham VK, Trappenburg MC, Kwang Lim W, Meskers CG et al (2019) Sarcopenia and its association with falls and fractures in older adults: a systematic review and meta-analysis. J Cachexia Sarcopenia Muscle 10:485–500 30993881 10.1002/jcsm.12411 PMC6596401

[45] Nielsen LM, Maribo T, Kirkegaard H, Bjerregaard K, Oestergaard LG (2020) Identifying elderly patients at risk of readmission after discharge from a short-stay unit in the emergency department using performance-based tests of daily activities. BMC Geriatr 20:217 32571229 10.1186/s12877-020-01591-y PMC7310017

[46] Alakare J, Kemp K, Strandberg T, Castrén M, Jakovljević D, Tolonen J et al (2021) Systematic geriatric assessment for older patients with frailty in the emergency department: a randomised controlled trial. BMC Geriatr 21(1):1–11 34215193 10.1186/s12877-021-02351-2 PMC8252275

[47] Dwyer R, Jachno K, Tran T, Owen A, Layton N, Collyer T et al (2024) Symptoms of depression and risk of emergency department visits among people aged 70 years and over. BMC Public Health 24(1):1–11 38317172 10.1186/s12889-024-17794-6 PMC10845391

[48] McCusker J, Bellavance F, Cardin S, Trépanier S, Verdon J, Ardman O (1999) Detection of older people at increased risk of adverse health outcomes after an emergency visit: the ISAR screening tool. J Am Geriatr Soc 47(10):1229–1237 10522957 10.1111/j.1532-5415.1999.tb05204.x

[49] Meldon SW, Mion LC, Palmer RM, Drew BL, Connor JT, Lewicki LJ et al (2003) A brief risk-stratification tool to predict repeat emergency department visits and hospitalizations in older patients discharged from the emergency department. Acad Emerg Med 10(3):224–232 12615588 10.1111/j.1553-2712.2003.tb01996.x

[50] Hoot NR, Aronsky D (2008) Systematic Review of Emergency Department crowding: causes, effects, and solutions. Ann Emerg Med 52(2):126–136 18433933 10.1016/j.annemergmed.2008.03.014 PMC7340358

