# Supplementary material for: Risk screening by the emergency medical services identifies older patients at risk of emergency department readmission: a retrospective observational study
Source: Aging Clin Exp Res. 2025 Mar 1;37(1):59. doi: 10.1007/s40520-025-02942-8 (PMC11870983; doi:10.1007/s40520-025-02942-8)
Supplement: Supplementary file 1 — Supplementary Material 1 [file 40520_2025_2942_MOESM1_ESM.docx]

*Supplementary materials*

Title: Risk screening by the emergency medical services identifies older patients at risk of emergency department readmission: a retrospective observational study

Journal: Aging Clinical and Experiental Research

Authors: Saario E, Mäkinen M, Castrén M, Jämsen E.

Corresponding author: Eeva Saario, Department of Emergency Medicine and Services, Helsinki University Hospital and University of Helsinki, Helsinki, Finland & Prehospital Emergency Medical Services, Satakunta Wellbeing Services County, Pori, Finland, [eeva.saario@helsinki.fi](mailto:eeva.saario@helsinki.fi)

**
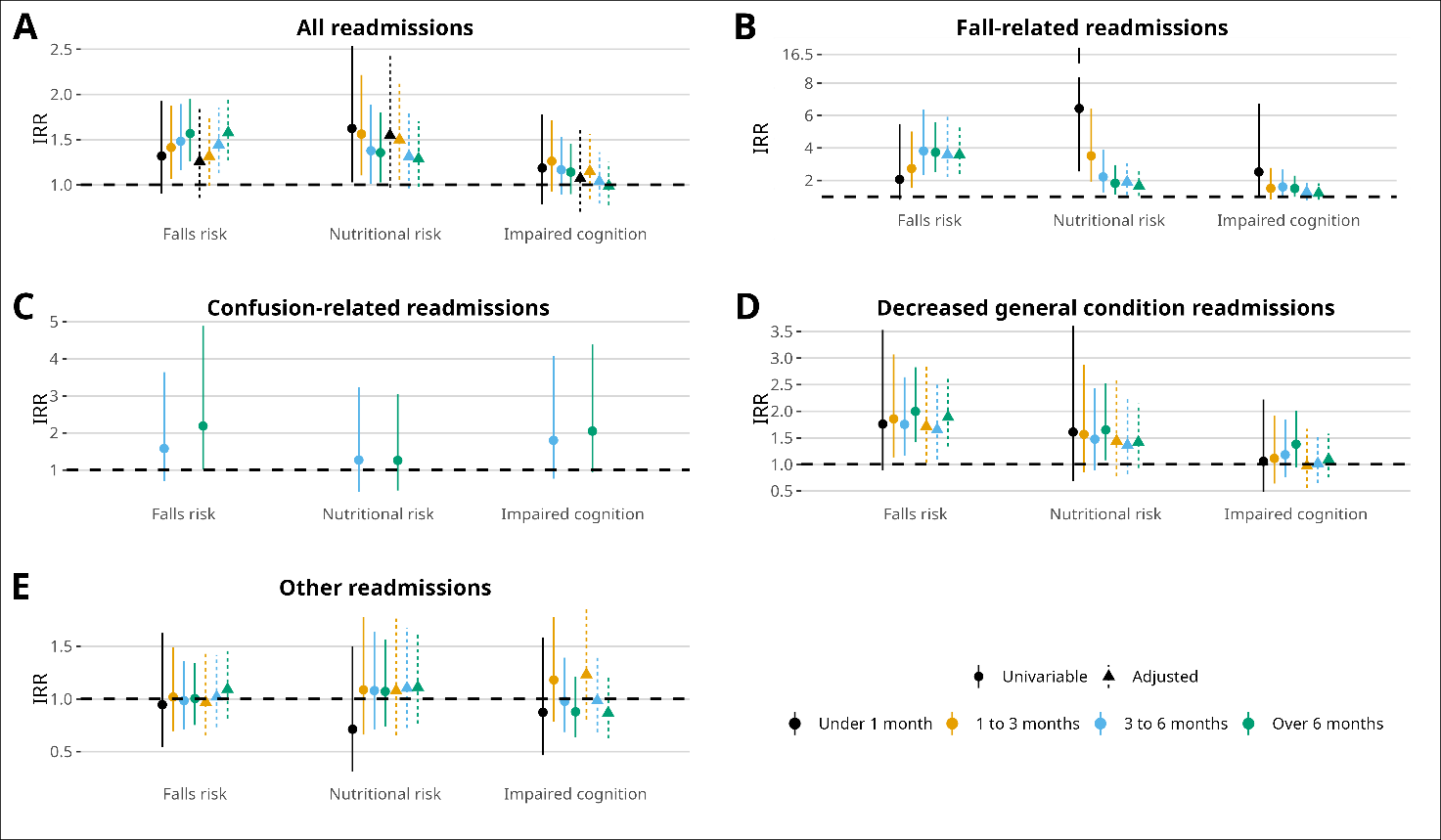
**

**Association of variables on all ED readmissions**

*Results presented in each time category as IRR (95% CI)

**Association of variables on fall-related ED readmissions**

* Results presented on each time category as IRR (95%CI)

Not enough units for multivariable analysis in first 3 months

**Association of variables on ED readmissions due to decreased general condition**

*Results presented in each time category as IRR (95% CI)

Not enough units for multivariable analysis in <1 month

**Association of variables on confusion-related ED readmissions**

*Results presented in each time category as IRR (95% CI)

Not enough units for univariate analysis in first 3 months of any multivariable analysis

**Association of variables on ED readmissions due to other reasons**

*Results presented in each time category as IRR (95% CI)

Not enough units for multivariable analysis in <1 month

**Association of variables on frequent user patients**

Values are median (IQR) or count (percentage in group). Tested using Mann-Whitney U tests (age, CCI) and Fisher’s tests (the rest).
